# Prostaglandin F-2α Stimulates The Secretion of Vascular Endothelial Growth Factor and Induces Cell Proliferation and Migration of Adipose Tissue Derived Mesenchymal Stem Cells 

**DOI:** 10.22074/cellj.2018.5026

**Published:** 2018-03-18

**Authors:** Abdolkhaleg Deezagi, Samira Shomali

**Affiliations:** Department of Molecular Medicine and Biochemistry, National Institute of Genetic Engineering and Biotechnology, Tehran, Iran

**Keywords:** Angiogenesis, Mesenchymal Stem Cells, Prostaglandin F2α, Scratching, Vascular Endothelial Growth Factor

## Abstract

**Objective:**

Tissue engineering today uses factors that can induce differentiation of mesenchymal stem cells (MSCs) into other cell types. However, the problem of angiogenesis in this differentiated tissue remains an unresolved area of research interest. The aim of this study was to investigate the effects of prostaglandin F-2α (PGF-2α) on the expression of vascular endothelial growth factor (VEGF) in human adipose tissue derived MSCs.

**Materials and Methods:**

In this experimental research, human adipose tissue was digested using collagenase. The isolated MSCs cells were treated with PGF-2α (up to 5 μg/ml) and incubated for 96 hours. Cell proliferation, secretion of VEGF and cell migration were spontaneously assayed by MTT, BrdU, ELISA, RT-PCR and scratching methods.

**Results:**

Cell growth at 1.0, 2.5, 5 µg/ml of PGF-2α was not significantly reduced compared to control cells, suggesting that these concentrations of PGF-2α are not toxic to cell growth. The results of the BrdU incorporation assay indicated that, in comparison to untreated cells, BrdU incorporation was respectively 1.08, 1.96, 2.0 and 1.8 fold among cells treated with 0.1, 1.0, 2.5 and 5.0 µg/ml of PGF-2α. The scratching test also demonstrated a positive influence on cell proliferation and migration. Cells treated with 1.0 µg/ml of PGF-2α for 12 hours showed the highest relative migration and coverage in comparison to untreated cells. Quantitative VEGF ELISA and RT- PCR results indicated an increase in VEGF expression and secretion in the presence of PGF-2α. The amount of VEGF produced in response to 0.1, 1.0, 2.5 and 5.0 µg/ml of PGF-2α was 62.4 ± 3.2 , 66.3 ± 3.7, 53.1 ± 2.6 and 49.0 ± 2.3 pg/ml, respectively, compared to the 35.2 ± 2.1 pg/ml produced by untreated cells.

**Conclusion:**

Stimulation of VEGF secretion by PGF-2α treated MSCs could be useful for the induction of angiogenesis in tissue engineering *in vitro*.

## Introduction

Research in the field of tissue engineering has made considerable progress in the last decade because of the rapid development of bioengineering and biotechnology. Research in the that field of tissue engineering has focused on the development of biological substitutes to restore and/or replace the function of damaged, diseased organs and tissues (1, 2). In recent years interest in studying mesenchymal stem cells (MSCs) and harnessing their unique differentiation capabilities for tissue engineering and regenerative medicine have increased (3, 4). MSCs are capable of self-renewal and multilineage cell differentiation under appropriate conditions. Due to these special characteristics, their availability in different tissues, high proliferation rate, long term viability and the lack of ethical and legal problems with their usage, they can be promising tools for cell replacement therapy (5, 6). 

One of the greatest challenges in tissue engineering today is angiogenesis and vascularization of new organs. Between different strategies, cell-based approaches have emerged as having particular promise (7). The use of endothelial cells to engineer vascularized tissues has been extensively investigated. This field of research has evolved with the discovery of endothelial progenitor cells, a subpopulation with high regenerative potential (8).

Angiogenesis is a key event in physiological and pathological processes. For this reason the inhibition and stimulation of angiogenesis constitute novel therapeutic strategies for several human diseases, including cancer (inhibition of tumor growth and metastasis), inflammation, cardiac hypertrophy, peripheral arterial disease, and ischemic heart diseases (wound healing and developmental progress) (9). Angiogenesis is closely regulated by growth factors such as vascular endothelial growth factor (VEGF) and intracellular signaling pathways (10).

A major stimulus for VEGF release is hypoxia which frequently occurs in the wound environment (11). VEGF and VEGF receptors, particularly VEGF receptor 2 (VEGFR2/Flk-1), are considered to constitute the key signaling system regulating endothelial cell proliferation and migration. Therefore, the suppression and/or induction of the VEGF signaling pathway is considered a potential strategy for tumor angiogenesis inhibition and/or tissue engineering and wound healing (12). VEGF-A is one of the biologically active factors secreted by stem cells. It is one of the important angiogenesis cytokines that not only has angiogenesis properties but can also increase auto crine production of several angiogenic proteins including angiogenin, interleukin 6 (IL-6), IL-8, transforming growth factor-ß1 (TGF-ß1), monocyte chemoattractant protein-1 (MCP-1) and matrix metallopeptidase-9 (MMP9) in several cell types (13, 14).

Remodeling of cytoskeletal elements, cell-cell recognition and reorganization are essential mechanisms for dynamic endothelial permeability and formation regulation. These processes are controlled by protein kinase signaling pathways and their related modulators (activators and inhibitors) (15). One of the sources of these angiogenic and vasculogenic cytokine natural modulators are the populations of mesenchymal cells which exist in different tissues. Many factors, such as Arachidonic acid, Prostaglandins, Cyclooxygenase modulators, Nitric Oxide, etc., can induce and stimulate these processes (16). 

Prostaglandins (PG) are products of the arachidonic acid metabolic pathway and synthesized by many tissues including vascular endothelial cells. The role of prostaglandins and their receptors in inflammation and regulation of vascular permeability is complicated. Although prostaglandins may be involved in the generation of acute lung inflammation, in part via vasodilatory effects, prostaglandin I2 (PGI2) and PGF-2α exhibit protective effects in the resolution phase of inflammation (17, 18). The aim of this work was to study the effect of PGF-2α in terms of the stimulation and production of VEGF by MSCs and its effect on cell proliferation and migration. We performed a multiple experiments to investigate the relationship between PGF-2α and VEGF expression MSCs. 

## Materials and Methods

### Isolation and culture of human adipose tissue-derived mesenchymal stem cells 

This experimental investigation was approved by the Institutional Review Board of the National Institute of Genetic Engineering and Biotechnology of Iran. Human adipose tissue was prepared after receiving informed consent from 3 healthy female volunteers who had been referred to the Sinai Shamiran clinic in Tehran for liposuction. Adipose tissue derived MSCs were isolated according standard methods as described by Lim et al. (19) and Yang et al. (20). Briefly, samples were washed with sterile phosphate buffer saline (PBS). Connective tissue surrounding the parenchyma was removed and samples were digested with 0.01% collagenase type I (Calbiochem-Merck-Bioscience, Germany) in PBS for 60 minutes at 37°C with gentle agitation. The enzyme activity was then neutralized by adding Dulbecco’s Modified Eagle Medium (DMEM)/10% fetal bovine serum (FBS) in a ratio of 1:1. After centrifugation for 10 minutes at 176.72 g and removal of the supernatant the cellular pellet was washed and then plated in T_75_ flasks which contained DMEM: F12 medium supplemented with 10% FBS and 1% penicillin/streptomycin (Calbiochem, Germany) and was put in an incubator with a humidified atmosphere containing 5% CO_2_ at 37°C. After 24 hours, the flasks were washed with PBS and their medium entirely changed. In this way non-adherent cells were removed. Adherent MSCs were then expanded by serial passage to improve the purity of the preparation and to generate a homogeneous cell population. The cells were detached with 0.25% trypsin-0.02% EDTA (Merck, Germany) and passaged at a ratio of 1:3 in every passage. MSCs were counted using a Neobar hemocytometer and viability was assessed using the trypan blue (Sigma, USA) exclusion test.

### Immunostaining of mesenchymal stem cells\ 

MSCs express different markers, such as vimentin, CD90, CD105, and CD44 (19). In this study the isolated MSCs were characterized through the expression of Vimentin after treatment with mouse anti-human Vimentin monoclonal antibody (Dako, Denmark) using immunocytochemical staining according to the procedure manual. In brief, first the cells were cytospun (1000 rpm, 5 minutes) in a cytospin centrifuge (Shandow, USA) and transferred as a monolayer onto glass slides. The cells were then fixed using formaldehyde (Merck, Germany) and incubated with mouse Anti-human Vimentin Ab (Dako, Denmark). After washing 3 times, peroxidase conjugated Rabbit-Anti Mouse Ab (Dako, Denmark) was added. Finally, color development was undertaken using Di-Amino Benzidine (DAB, Calbiochem, Germany) as substrate. 

### Treatment of adipose tissue-derived mesenchymal stem cells by prostaglandin F-2α 

During passages 3-8, the cells (2×10^4^ cells/cm^2^) were separately treated using PGF-2α (up to 5 µg/ml, Daru- Pakhsh Company, Iran) in 5 mL of DMEM medium containing 10% of FCS in 6-well plates (NUNC, Denmark). The cells were incubated at 37°C for 96 hours. The supernatant and pellets from the treated cells were then collected separately. The effect of PGF-2α on cell growth and cell proliferation was assessed by cell counting, and the MTT and BrdU cell proliferation assays as described below.

### MTT assay

Cytotoxicity was assessed using the MTT assay according to standard protocols. The MTT assay is commonly used to assess cell proliferation and viability by measuring the reduction of yellow MTT by mitochondrial dehydrogenases in viable cells. 10^4^ cells per well were seeded in 96-well microplates in 100 µl of complete culture medium and treated with PGF-2α (up to 5 µg/ml) for 96 hours. Following treatment, the MTT reagent was added (10 µl/well) and the cells were incubated at 37°C for an additional 4 hours. Finally, 150 µl of dimethyl sulfoxide (DMSO, Merck, Germany) was added to dissolve the formazan crystals and absorbance was read in a microplate ELISA reader at a wavelength of 540 nm. The viable cell number was directly proportional to the production of formazan. 

### BrdU assay

BrdU can be incorporated into the newly synthesized DNA of replicating cells and is commonly used to assess cell proliferation. As for the MTT assay, 104 cells per well were separately seeded in 96-well microplates in 100 µl of complete culture medium and treated with PGF-2α (up to 5 µg/ml) for 96 hours. After that the cells were assayed for BrdU incorporation using the BrdU Cell Proliferation Kit according the procedure manual (Roche, Germany). The basis of the method and full details have been described in our previous work (21). Briefly 10 µl of BrdU was added to each well and incubated for 24 hours. Then cells were washed 3 times and fixed. The incorporation of BrdU was detected by specific Anti BrdU Abs (Roche, Germany) using immunologic methods and final color development detected using an ELISA reader at a wavelength of 450 nm.

### Scratching assay

Cell migration was measured using the wound healing assay as described by Staton et al. (22). Briefly, equal numbers of cells (5×10^5^) were plated and kept overnight in 6-well plates. After that the monolayer of cells was wounded by manual scratching with a micro-pipette tip head and the cells treated with different concentration of PGF-2α. The cells were then photographed using a phase contrast microscope (Nikon) (0 hour point). The cells were cultured in complete growth medium and incubated. Matching wound regions were photographed after different time intervals up to 96 hours.

The photographs of the cells were analyzed using aCell science software program. The distance betweenthe two sides of scratch layers of images taken frommigration of all concentrations at specified times wasdetermined by the Cell science software. The averagedistance in each photograph was calculated and thepercentage difference for each concentration at the same specified time was calculated and compared. These results are presented as a graph generated by Excel program statistical software. 

### Reverse transcription polymerase chain reaction for vascular endothelial growth factor expression

At different time intervals, PGF-2α treated cells were collected. Total RNA was extracted using the TriPure (sina Gene kit, Iran), following the manufacturer’s instructions. The concentration and purity of total RNA in each sample was determined by the A260/A280 ratio using a Beckman DU-70 spectrophotometer (Beckman Instrument Inc., Fullerton, CA, USA). The integrity of RNA was confirmed by electrophoresis on agarose ethidium bromide gel. First-strand complementary DNA (cDNA) was synthesized from 1 µg of total RNA using the murine Moloney leukemia virus, reverse transcriptase (RT), and oligo-dT primer (MBI Fermentas, St. Leon-Rot, Germany), according to the manufacturer’s instructions. *hVEGF-cDNA* and *ß-actin* cDNA were amplified by the primers listed in Table 1. 

The thermal cycling conditions for amplification of the *hVEGF* (250 bp) and *ß-actin* (530 bp) fragments has been described by us previously (23). Briefly, the conditions were as follows: 95°C for 5 minutes, followed by 30 cycles at 95°C, 30 seconds; 60°C, 30 seconds; 72°C, 30 seconds; and 72°C for 5 minutes. The polymerase chain reaction (PCR) products were separated on a 2 % (w/v) agarose gel (using 0.59 TBE buffer) and visualized using ethidium bromide (Sigma-Aldrich, St. Louis, MO, USA) staining. The amount of PCR product was calculated using an external (*ß-actin*) standard curve and Flourchem SA software. All values were normalized on the basis of *ß-actin* expression in the corresponding samples. Specific primers for the genes examined were based on their NCBI/Primer-BLAST sequences.

**Table 1:** The primer sequences of the sense and antisense for reverse transcription-polymerase chain reaction (RT-PCR) of VEGF and β-actin genes


Sequencedefinition	Annealingtemp (˚C)	Sequence primer (5ˊ-3ˊ)

VEGFA	60	F: CCATGAACTTTCTGCTGTCTT
		R: ATCGCATCAGGGGCACAC
β-actin	59.3	F: ACAGAGCCTCGCCTTTGCCG
		R: CTTGCTCTGGGCCTCGTCGC


### Quantitative vascular endothelial growth factor ELISA assay

The amount of VEGF in cell supernatants was measured using a commercial Calbiochem hVEGF ELISA kit (Calbiochem-Merck-Bioscience, Germany) according the procedure manual. The human VEGF ELISA kit is a "sandwich" enzyme immunoassay employing monoclonal and polyclonal antibodies. Quantitation is achieved by construction of a standard curve using known concentrations of human VEGF proteins. The procedure has been explained in detail previously (23).

### Statistical analysis

All statistical analyses were performed using Microsoft Office Excel 2010 software. The data are expressed as mean ± SD. The significance of differences between groups was determined using the Tukey HSD test or One-Way ANOVA. A P<0.05 was considered to denote statistical significance. 

## Results

### Isolation, culture and characterization of mesenchymal stem cell

**Fig.1:**
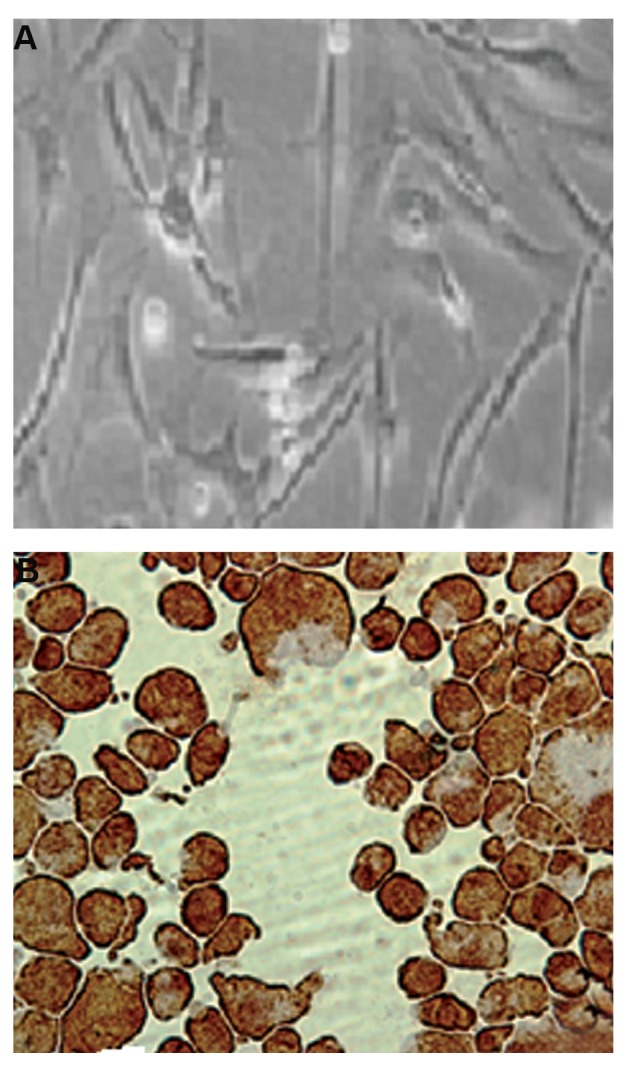
Human mesenchymal stem cells (MSCs) were isolated from human adipose tissue and culture and characterized by Vimentin immunostaining as described in the methods. A. The morphology of the cells fully stretched, spindle-shaped, and without appendages which are pressed together and B. The immunocytochemistry staining of the cells by human Vimentin monoclonal antibody; the cultured cells express vimentin which indicates that they are mesenchymal cells (magnification: ×400).

Human MSCs were isolated from human adipose tissue by digestion of tissue using collagenase type I as described in the materials and methods. More than 80% of the cells were viable after isolation. MSCs were sub-cultured and passaged at 4 day intervals. After further time, the cells gradually became larger in size and were observed as elongated with long appendages. Their morphology was changed into a layer of cells fully stretched, spindle- shaped, and without appendages which are pressed together (Fig .1A). The immunocytochemistry staining of the cells by human Vimentin monoclonal antibody is shown (Fig .1B). As the figure shows, the cultured cells express vimentin indicating that they are mesenchymal cells.

### Effect of PGF-2α on cell growth and proliferation

**Fig.2:**
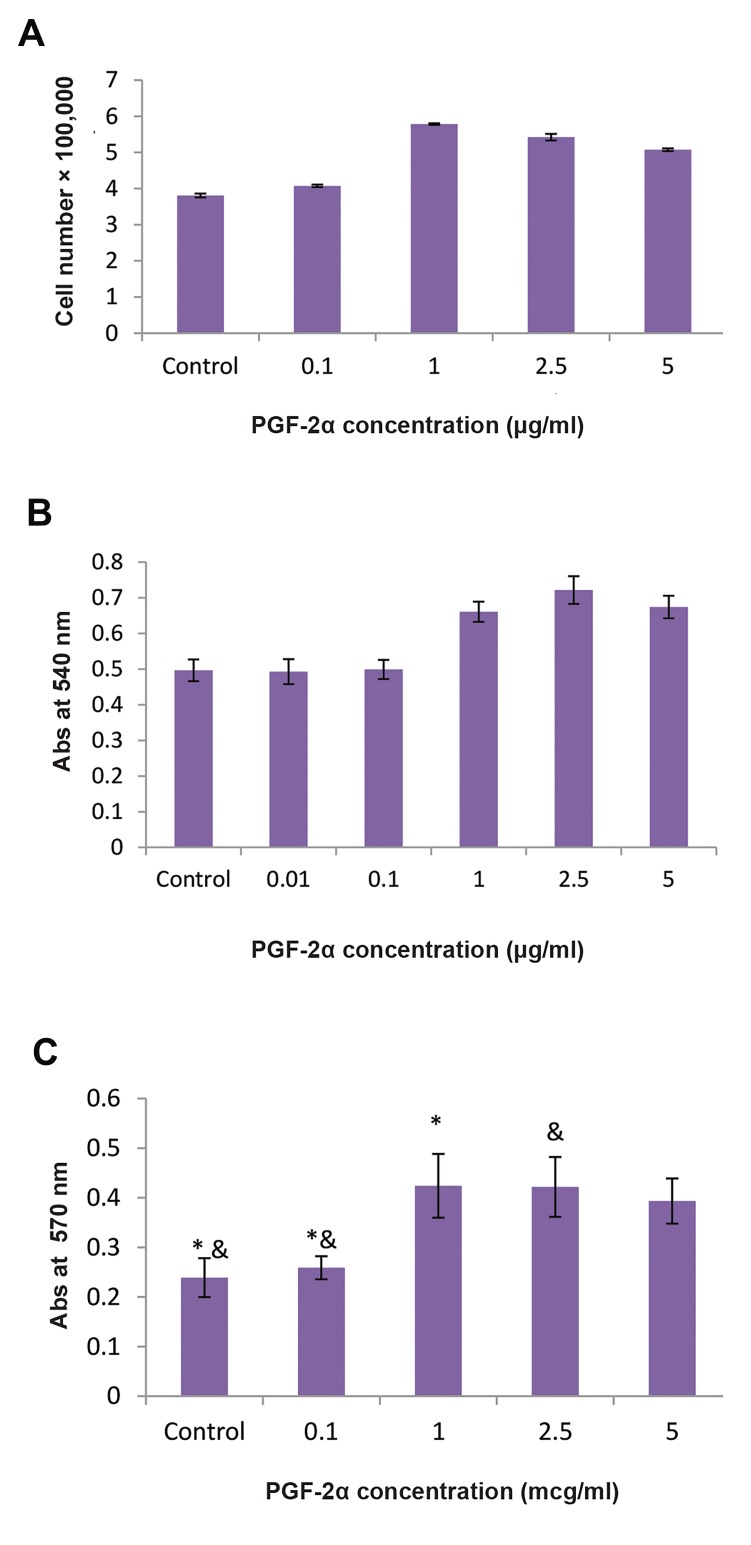
Proliferation and growth of mesenchymal stem cells (MSCs) after treatment by PGF-2α. The cells were grown in RPMI medium in the absence or presence of PGF-2α for 96 hours as described in materials and methods. Then, control and treated cells were collected: A. Total cell number was determined using a hemocytometer. The results are mean ± SEM. for three separate experiments, B. The MTT cell proliferation assay, and C. The BrdU cell proliferation assay. The results are mean ± SEM for three separate experiments. *; P<0.01 compared to 1 and 2.5 µg/ml of PGF-2α in untreated cells and &; P<0.01 Compared to 2.5 µg/ml of PGF-2α in untreated cells.

Comparing the growth and proliferation of cells treated with PGF-2α and control cells after 96h showed that cell growth, in terms of number of cells counted using a hemocytometer, was significantly greater at concentrations of 1, 2.5, 5 µg/ml PGF-2α (P<0.05) than for controls (Fig .2A). The effect of different concentrations of PGF-2α on the growth of MSCs was also evaluated using an MTT assay. In fact, natural conditions for the proliferation of MSCs at best condition of the culture medium has a 10% FBS. Because implementation of the MTT assay at lower concentrations of FBS was not executable due to the complete stop in growth of MSCs. The results showed that growth of cells treated with 0.1, 1.0, 2.5, 5.0 µg/ ml of PGF-2α had a significant increase compared to the control sample (Fig .2B). It was also observed that the mentioned three levels of concentration had a significant statistical difference with the concentration of (0.1 µg/ml). The growth of cells treated with (0.1 µg/ml) concentration of PGF-2α had not a significant growth compared to the control group. In all, our results demonstrated the non-toxicity of PGF-2α on the growth of MSCs.

Results of the BrdU assay showed that the growth of cells treated with 1.0 and 2.5 µg/ml of PGF-2α was significantly increased compared to the control group (Fig .2C). The absorbance for untreated control cells was 0.231 ± 0.051 and for cells treated by 0.1, 1.0, 2.5 and 5.0 µg/ml of PGF-2α was 0.248 ± 0.037, 0.453 ± 0.081, 0.461 ± 0.075 and 0.413 ± 0.058 respectively. These data indicate that BrdU incorporation was 1.08, 1.96, 2.0 and 1.8 fold respectively in treated cells in comparison to untreated cells. It was also observed that growth at the last three concentrations was significantly different from growth at the (0.1 µg/ml) concentration. The growth of cells treated with 0.1 µg/ ml concentrations of PGF-2α did not have a significant effect compared to the control group. As for the tests above, the overall results of studying the effect of PGF2α showed no toxic effects on the proliferation of MSCs.

### The effects of PGF-2α on migration of mesenchymal stem cells in scratching method

**Fig.3:**
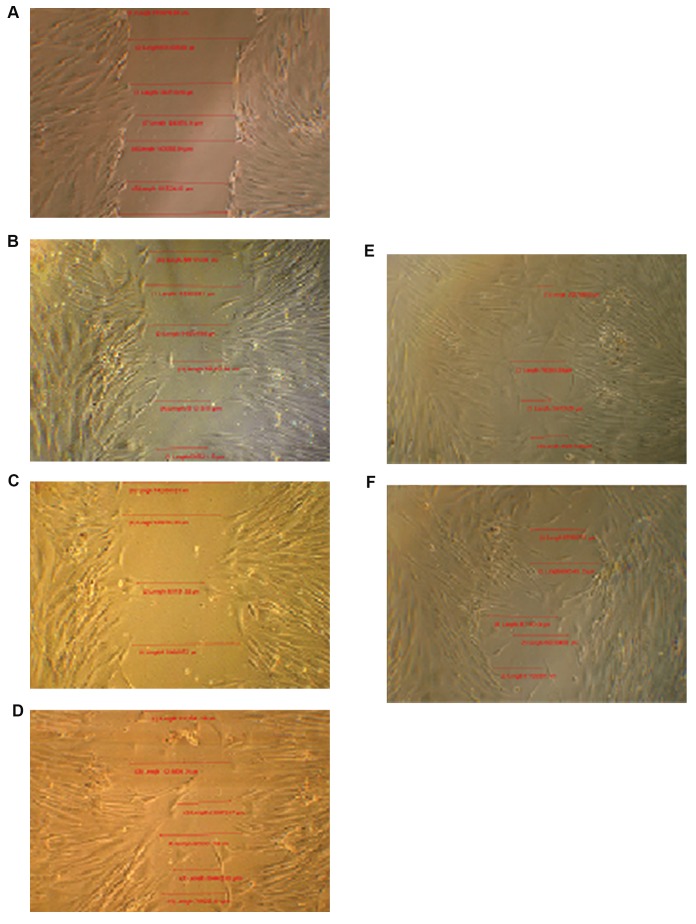
Migration of mesenchymal stem cells (MSCs) after treatmentby PGF-2α (up to 5 µg/ml) after 0, 6, 12, and 24 hours as describedin materials and methods. The MSCs were grown in RPMI medium inthe absence or presence of migration inducing agent (PGF-2α). Cellswere assessed for migration by the scratching method. The distanceof migration and relative surface coverage area were calculated bythe program which is described in methods. A. The morphology and photographs of the cells at time zero, B-F. Show cells treated with PGF-2α (0.0, 0.1, 1.0, 2.5 and 5 µg/ml) 12 hours after scratching(magnification: ×400), and G. The results are mean ± 1.0 SEM. for three separate experiments.

To assess the effect of different concentrations of PGF-2α on the proliferation and migration of MSCs scratching was performed as described in the materials and methods. The morphology and photographs of the cells are shown (Fig .3A). The photographs of the cells were analyzed and calculated as described in materials and methods. The results were presented as a graph using statistical analysis (Fig .3B). Cells treated with 1.0 µg/ml of PGF-2α for 12 hours showed the highest relative migration and coverage in comparison to untreated cells. Statistical analysis of the results A indicated that treatment of the cells by 1.0, 2.5, 5.0 µg/ml of PGF-2α had significant effects in comparison to the untreated cells (P<0.05), but 0.1 µg/ml PGF-2α was not significantly different compared to untreated cells. 

### The effect of PGF-2α on the expression and secretion of vascular endothelial growth factor 

**Fig.4:**
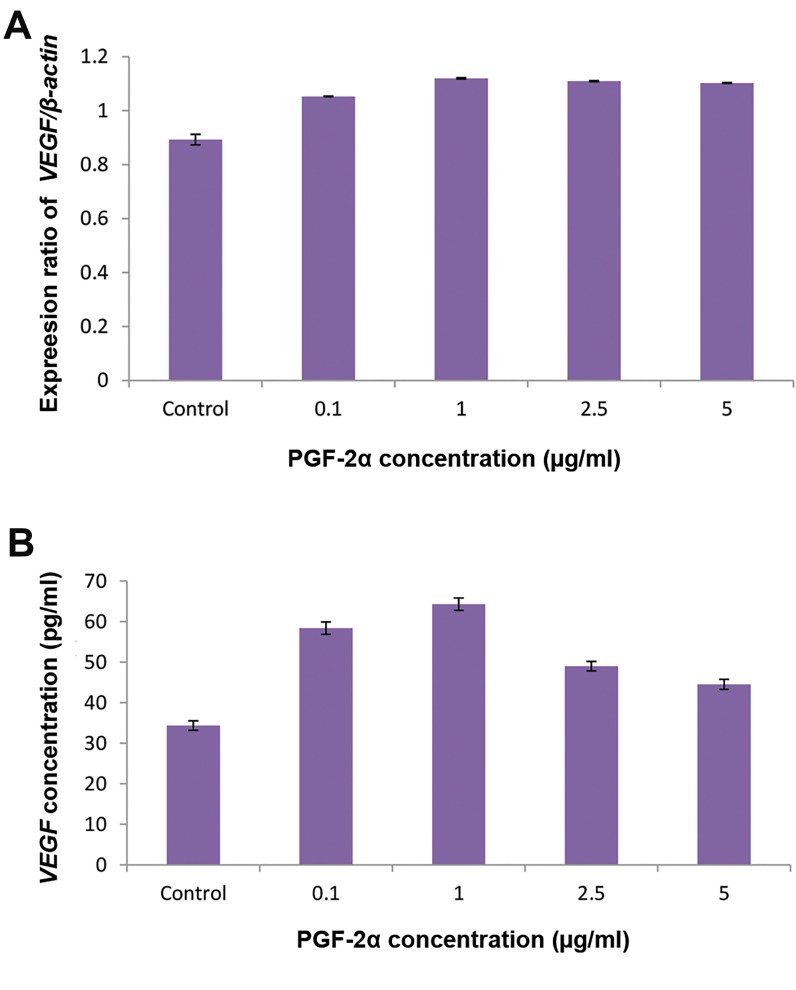
Changes in VEGF gene expression during the treatment of mesenchymal stem cells (MSCs) by PGF-2α (up to 5 µg/ml). MSCs were incubated with PGF-2α (up to 5 µg/ml) 96 hours as described in materials and methods. A. Total RNA was extracted from untreated and PGF2α treated cells and analyzed by RT-PCR for VEGF gene expression. ß-actin served as an internal housekeeping gene control. The results are mean ± SEM. for three separate experiments and B. The supernatant of the untreated and PGF-2α treated cells were collected and measured by quantitative human VEGF ELISA kit as described in the materials and methods.

We examined the expression of *VEGF* genes and *ß-actin*, which was used as an internal housekeeping control gene, in cultured MSCs treated with PGF-2α and untreated control cells as described in materials and methods. The ratio of each band of *VEGF* gene was calculated vs. *ß-actin* gene. The ratio of each band of each *VEGF* gene vs. the *ß-actin* gene was calculated and the results are presented (Fig .4A). 

Secretion of VEGF by PGF-2α treated cells was measured in the cell supernatant using an ELISA, as described in the materials and methods. The concentrations of VEGF were calculated as described in methods (Fig .4B). The amount of VEGF was 35.2 ± 2.1 for untreated cells and 62.4 ± 3.2 , 66.3 ± 3.7, 53.1 ± 2.6 and 49.0 ± 2.3 pg/ml for cells treated with 0.1, 1.0 , 2.5 and 5.0 µg/ml PGF-2α respectively. The results show that 0.1, 2.5, 5.0 µg/ml concentrations do not significantly increase VEGF secretion, but a concentration of 1.0 µg/ml produced a significant increase; approximately 2-fold compared to the untreated control.

## Discussion

This work used human MSCs isolated from liposuction fat. This tissue is easily and routinely available in large quantities and its cell efficiency is much higher than that of bone marrow tissue. Regardless of the volume of the original liposuction sample the MSC yield was consistent and represented 0.0005% of total cells. MSCs isolated from adipose tissue show a high proliferative capacity in culture medium without losing their morphological characteristics. Proliferation and growth of these cells in the presence of PGF2α were measured with BrdU and MTT assays, because in lesser amounts of serum, MSCs stopped growing and the test was not actually applicable. In this case, MSCs are able to secrete several growth factors such as VEGF, which plays a major role in inducing division of these cells. 

*VEGF* expression by MSCs after treatment with PGF-2α showed increased expression of this gene in comparison with the control sample. Comparing the tests, it seemed that PGF-2α effectively promotes the growth and proliferation of MSCs, and increases the influence of PGF-2α on the expression of the *VEGF* gene in these cells. Comparing all the tests, the highest and best response was observed for a concentration of 1 µg/ml PGF2α. 

The results of this and other similar studies indicate that PGF-2α seems to be in the category of substances capable of increasing angiogenesis factors in MSCs. This means it can act as a useful factor in angiogenesis in the tissue structures used in tissue engineering and help in angiogenesis inhibition in the case of angiogenesis dependent diseases (24). 

Different studies have confirmed that cultivation of stem cells is an important tool for the treatment of several types of malignancies. These cells have high potential for use in regenerative medicine as well as cell therapy and gene therapy for tumors and cancers which are in research and development phase of clinical trail. It has been suggested that the effects mediated by MSCs can be attributed to bioactive factors to the extent they are secreted by these cells in their target areas (25, 26).

MSCs provide a suitable source of cells for tissue engineering and are particularly important in pioneering methods for the long-term survival and function of tissue structures. The recognized ability of these cells to produce various angiogenesis factors, such as VEGF, and the advantage of using these cells in cell therapy, including the stimulation of the immune system, the lack of tumorigenic properties and their plasticity, or cross- differentiation has highlighted the importance of studying these cells (27).

Our results indicate that PGF2α may be able to improve the collaboration between endothelial cells and MSCs by improving the performance of MSCs in angiogenesis through increasing the production of factors such as VEGF in those cells. Angiogenic factors are released into the environment by MCSs and stimulate different types of normal cells, such as capillary endothelial cells near the tumor. These cells break down their base membranes, which support the endothelial cells, and, by detaching themselves from the neighboring cells and entering the extracellular matrix, they migrate towards the mass of the tumor. VEGF acts as a mitogen for vascular endothelial cells derived from arteries, veins and lymphatic vessels and increases angiogenesis. Studies also show that VEGF together with hypoxia inducing factor-1 (HIF1) can cause vascular permeability as measured by the vascular permeability factor (VPF). Increase in vascular permeability is a critical step in angiogenesis of tumors and wounds. According to this theory, the main function of VPF/VEGF in angiogenesis is increasing the leakage of plasma proteins. This leads to the formation of fibrin gel outside the vessels which is a substrate for the growth of endothelial and tumor cells (28, 29).

## Conclusion

Angiogenesis stimulation methods which induce angiogenesis and vascularization in tissue engineering are important for treating diseases associated with angiogenesis. Results of this work indicate that, stimulation of the VEGF secretion by MSCs could be useful for induction of angiogenesis in tissue engineering *in vitro*. 
